# Reproductive and Obstetric Factors Are Key Predictors of Maternal Anemia during Pregnancy in Ethiopia: Evidence from Demographic and Health Survey (2011)

**DOI:** 10.1155/2015/649815

**Published:** 2015-08-31

**Authors:** Taddese Alemu, Melaku Umeta

**Affiliations:** ^1^Center of Food Science and Nutrition, College of Natural Sciences, Addis Ababa University, P.O. Box 1196, Addis Ababa, Ethiopia; ^2^Department of Biochemistry, College of Medical Sciences, Addis Ababa University, P.O. Box 1196, Addis Ababa, Ethiopia

## Abstract

Anemia is a major public health problem worldwide. In Ethiopia, a nationally representative and consistent evidence is lacking on the prevalence and determinants during pregnancy. We conducted an in-depth analysis of demographic and health survey for the year 2011 which is a representative data collected from all regions in Ethiopia. Considering maternal anemia as an outcome variable, predicting variables from sociodemographic, household, and reproductive/obstetric characteristics were identified for analyses. Logistic regression model was applied to identify predictors at *P* < 0.05. The prevalence of anemia among pregnant women was 23%. Maternal age, region, pregnancy trimester, number of under five children, previous history of abortion (termination of pregnancy), breastfeeding practices, and number of antenatal care visits were key independent predictors of anemia during pregnancy. In conclusion, the level of anemia during pregnancy is a moderate public health problem in Ethiopia. Yet, special preventive measures should be undertaken for pregnant women who are older in age and having too many under five children and previous history of abortion. Further evidence is expected to be generated concerning why pregnant mothers from the eastern part of the country and those with better access to radio disproportionately develop anemia more than their counterparts.

## 1. Introduction

Maternal death continues to be a major health and development concern globally, particularly in the developing world [1, 2]. In 2013 alone, there were an estimated 289,000 maternal deaths (210 deaths per 100,000 live births) across the globe, of which the sub-Saharan Africa region accounting for 62% (179,000) of these. During the same period, the mortality rate in developing regions (230) was 14 times higher than in developed regions (16) whilst the sub-Saharan Africa recorded the highest (510) regional MMR [2]. Astonishingly, over the 99% of these annual deaths occurring in developing countries are avoidable, as the healthcare solutions to prevent or manage complications are well known.

Regarding the major causes of maternal death, about 73% of all deaths between 2003 and 2009 were due to direct obstetric causes. Haemorrhage (27.1%), hypertensive disorders (14.0%), sepsis (10.7%), abortion (7.9%), and embolism accounted for the majority of these direct causes. On the other hand, the indirect causes contributed to over 27.5% of the maternal deaths globally and 28.6% in sub-Saharan Africa [1].

Anemia is one of the leading indirect causes of maternal mortality and it is the most common and intractable nutritional problem globally [3]. Although easily preventable and treatable, it is one of the most serious threats to the health of children and a factor in maternal mortality. In pregnant women, anemia results in an increased risk of premature delivery and low birth weight. It is also known to be an important factor in maternal death, the poor cognitive development of children, and decreased work capacity of the mother. It also decreases the health and energy of approximately 500 million women and leads to approximately 50,000 deaths in childbirth each year [4].

The World Health Organization (WHO) defines anemia as hemoglobin concentrations that are below recommended thresholds [3, 5]. The main causes of anemia are dietary iron deficiency; infectious diseases such as malaria, hookworm infections, and schistosomiasis; deficiencies of other key micronutrients including folate, vitamin B12, and vitamin A; or inherited conditions that affect red blood cells (RBCs), such as thalassaemia [6].

The prevalence of anemia during pregnancy is quite high (42%) globally and above 57.1% in Africa, signifying it as a severe public health problem in the region [3]. In Ethiopia, even if the situation seems better, the latest EDHS estimated that above 22% of women during pregnancy were found anemic [7]. On the other hand, some cross-sectional localized studies conducted at various regions of the country demonstrated that the prevalence of anemia among women of the reproductive age group in general and pregnant women in particular ranged from as low as 16.6% in the north [8] to a modest (33.2%) level in the south [9] and high up to 43.9% [10] in the eastern parts of the country.

On top of variation in the prevalence rate, the very important aspect of these local studies stipulating further investigation is the fact that none of them presented with conclusive and consistent findings on the determinants of anemia during pregnancy. A number of dissimilar and mutually exclusive determinant factors were identified in the studies. For instance, the northern study heightened hookworm infestation and HIV infection [8], whereas daily chewing of khat, restrictive dietary behavior, parity levels, and pregnancy trimesters were identified by the eastern study. Another relatively representative nationwide study [11] conducted in nine regions of the country identified other factors like chronic illnesses and deficiency of iron/folic acid as key determinants of anemia during pregnancy [11].

Generally, even if these studies have given important clues to policy makers, programmers, and other stakeholders, they mainly lack consistency and representatives to be used for national level policy making and programming by concerned bodies. Therefore, these in-depth analyses of the latest (2011) EDHS provide in-depth and explicit results on the prevalence and key proximate determinants of maternal anemia during pregnancy in Ethiopia.

## 2. Methods

### 2.1. Data Source

This study uses data from the Ethiopian Demographic and Health Survey (EDHS) conducted in 2011. The survey was conducted with nationally representative samples from all of the country's regions. The details of the sample design, including the sampling framework and sample implementation, and response rates are provided in the respective EDHS reports (http://www.measuredhs.com/).

In the DHS, there are three core questionnaires (household, women's, and male questionnaires) and nine recode files. This way of recoding is done because of two outstanding reasons: to define a standardized file that would make cross-country analysis easier and to compare data with the World Fertility Surveys (WFS) to study trends [12]. The recode files have five main and two additional digits. The first two digits of the file name correspond to the country code (e.g., ET for Ethiopia). The next two digits identify the unit of analysis (IR—women, KR—children, etc.). The fourth digit identifies the DHS phase. The fifth digit identifies the data release number and the last two digits identify whether it is a rectangular (RT) or flat (FL) file; for the hierarchical file they are left blank.

In our current analyses, we used ETIR61FL.SAV recode data files for prevalence and analyses of determinant factors. This means that we used the 2011 IR (women with completed interviews) EDHS data to describe the prevalence and determinants of anemia during pregnancy in Ethiopia.

### 2.2. Study Variables

The dependent variable is maternal anemia during pregnancy. According to the WHO and International Nutritional Anemia Consultative Group (INACG) [5, 13] anemia during pregnancy, after adjusting for altitude, is defined as a hemoglobin concentration of 11.0 g/dL and hemoglobin levels of 10–10.9 g/dL, 7–9.9 g/dL, and less than 7.0 g/dL were considered as mild, moderate, and severe anemia, respectively. Other cutoff points of hemoglobin concentration specific to trimesters of pregnancy are suggested by the International Nutritional Anemia Consultative Group (INACG) and others.

The selection of potential predictors of maternal anemia during pregnancy in this study is based on the literature and the availability of variables from the EDHS data sets on these potential predictors. An attempt is also made to test all potentially relevant variables existing in the EDHS data sets before concluding dropping of them; therefore, all variables which showed a statistical significance or some sort of trend during bivariate analyses are included into the analyses.

These potential predicting variables are categorized into three groups: sociodemographic characteristics, household variables, and maternal reproductive characteristics.

*Sociodemographic Variables*. These groups of indicators consist of both maternal and paternal (husbands') characteristics. Among the maternal characteristics, maternal age, urban/rural residence, region, educational status, and literacy level are included.

*Household Variables*. In this group, we included presence or absence of key household variables like electricity, radio, and television. Other variables included into this category are type of toilet facility (whether the household uses improved or nonimproved type of toilet) and sources of drinking water.

*Maternal Reproductive and Obstetric Variables*. Attributable to the nature of the study and availability of evidence by a wide range of literatures, a number of variables are included into this category as compared to the other two. Accordingly, pregnancy trimester, number of births in the last five years and last year, whether the current pregnancy is wanted or not, history of abortion (pregnancy termination), practice of breastfeeding, timing of first ANC visit, and number of ANC visits are all included.

### 2.3. Data Analysis

This study employed a three-stage analysis. In the first stage, univariate and bivariate analyses of the level (prevalence) of maternal anemia during pregnancy by the three categories of variables mentioned were calculated using chi-square, ANOVA, and Student's *t*-test.

In the later stage, binary logistic regression analyses of each variable to determine the crude odds ratio of each identified variable with maternal anemia were calculated. After making an appropriate selection of variables having a strong association with the dependent variable, we moved to the third stage of analyses of multivariate logistic regression analyses of all variables found statistically significant. SPSS version 20 software was used to run all stages of the analyses.

### 2.4. Data Quality Assessment

The data quality assessment report highlights its findings on misreporting, omission, and digit preference, which are common data quality problems observed in surveys and censuses in developing countries. Over all, the assessment shows that the problems do not exist to the extent that might challenge the quality of the conclusions of this study.

## 3. Results

**Figure 1:**
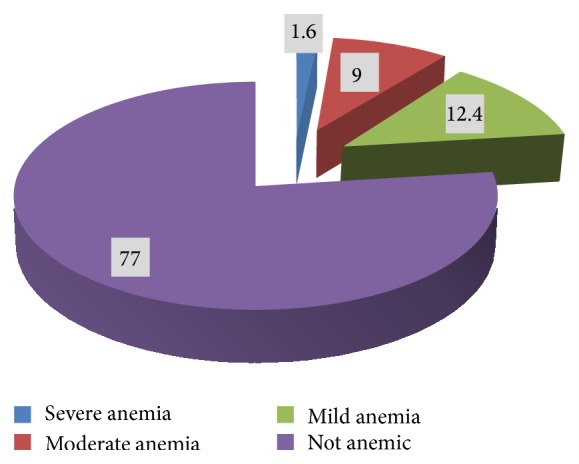
Prevalence and severity of anemia among pregnant mothers in Ethiopia, EDHS 2011.

A total of 1,212 pregnant women were included into the initial analyses which is 10.2% of the total women of the reproductive age group from whom data was collected in the same survey. The overall prevalence of anemia in this group was found to be around 23%, varying from 12.4% milder forms to 9% and 1.6% moderate and severe forms, respectively (Figure 1 and Table 1).

In terms of maternal age, the lowest (12.5%) and the highest (30%) prevalence of anemia were seen among teenagers (15–19) and older (40–45) women, respectively. Women in the latter group had a 3.43 more added risk of developing anemia during pregnancy as compared to the reference age group (15–19) (AOR, 3.43; 95% CI, 1.04–11.28). Pregnant women aged (30–34) and (20–24) had the second highest (27.1%) and lowest (19.1%) prevalence of anemia, respectively (*P* > 0.05) (Table 1).

Pregnant women from urban setting had a nonsignificant but higher (26.9%) prevalence of anemia as compared to the rural counterpart (22.7%) (AOR, 0.80; 95% CI, 0.43–1.48). In the same way, the prevalence of anemia among pregnant women across different regions in the country ranged from as low as 0% in Addis Ababa followed by 10.6% and 21.6% in SNNPR and Tigray regions. It was also as high as 60%, 51.9%, and 38.9% in regions like Dire Dawa, Somali, and Afar, respectively (all of these regions are Muslim community dominated and are located in the eastern part of the country). The variation was significant for SNNPR (AOR, 0.41; 95% CI, 0.21–0.82) and Somali regions (AOR, 3.82; 95% CI, 1.71–8.52).

Considering the educational and literacy characteristics of mothers, even if a statistically significant difference was observed during the bivariate logistic regression analyses, none of these appeared in the final multivariate regression model. Yet, descriptive analysis shows that women with no formal education and those trained to tertiary level had a higher, 25% and 33.3%, prevalence of anemia during pregnancy, respectively. Literacy level showed a nonsignificant but a linear declining trend of anemia prevalence; that is, the higher the woman is literate, the lower is the risk of developing anemia during pregnancy (*P* > 0.05) (Table 1).

Table 2 shows that, among the household variables analyzed, all were found not to be associated with anemia of a pregnant women, but radio ownership. Pregnant women who reported lack of access to household variables like electricity, television, and safe water reported almost similar (23.4%–23.7%) prevalence of anemia which is lower than their counterparts. Contrarily to expectations and biological plausibility, however, pregnant women, who were supposed to have a lesser prevalence as they own/have access to radio and improved sanitation, reported a higher 27.2% (AOR, 1.41; 95% CI, 1.01–1.88) and 25.3% (AOR, 0.81; 95% CI, 0.45–1.43) prevalence of anemia than their counterparts, respectively (Table 2).

Regarding the obstetric factors analyzed, almost all of these showed a strong association (increased risk or protective benefit) with the outcome variable of interest, amongst which, pregnancy trimester, number of under five children, previous history of abortion (termination of pregnancy), practice of exclusive breastfeeding, and number of antenatal care visits showed a statistical significance association.

Pregnant women in their second and third trimesters of pregnancy had a 17-fold higher risk of developing anemia compared to those in the first and second trimesters (AOR, 17.05 and 17.71; 95% CI, 3.69–78.8 and 3.76–83.27), respectively. In the same way, pregnant women who have two or more under five children (AOR, 0.39; 95% CI, 0.18–0.84) have previous history of pregnancy termination (AOR, 2.63; 95% CI, 1.17–5.92), who have never breastfed or continued breastfeeding to present pregnancy (AOR, 7.64 and 4.05; 95% CI, 2.28–25.65 and 1.61–10.91), and those who had adequate (3-4) and/or frequent antenatal care visits (AOR, 0.49 and 0.36; 95% CI, 0.19–0.98 and 0.08–0.64), respectively, had a statistically significant higher (increased) or lower risk of developing anemia during pregnancy (Table 3).

## 4. Discussion

This in-depth analysis of the latest (2011) Ethiopian demographic and health survey shows that the prevalence of anemia among pregnant women (23%) in the country is comparable to the nonpregnant women of reproductive age group (22%) and to the level of moderate public health problem. The prevalence is also lower than several other local cross-sectional studies [9, 14, 15] and previous EDHS reports [16, 17]. This shows a declining trend of anemia in the country. On the other hand, the value is far below regional and international estimates [18–20]. This shows the results of the current study should be interpreted cautiously as the data was collected for comprehensive survey of health and demographic events and the samples included in this study might not be good representative of pregnant mothers.

On the other hand, previous similar surveys [16, 17, 21], cross-sectional studies, and other reports [9, 14, 15] in the country have revealed almost similar trend. Studies of food analysis in the country have shown that, if not for bioavailability, most staple diets are rich in iron [22, 23] which might has contributed to lesser prevalence of anemia compared to the predictions and other countries of the sub-Saharan African region where the prevalence of anemia is the highest in the world.

In this study, older women were at a higher risk of anemia compared to teenagers (AOR, 3.43; 95% CI, 1.04–11.28) and the prevalence also increased with age (*P* < 0.05). This is consistent with previous local studies [8, 14]. On the other hand, pregnant mothers from some regions in the country had relatively higher prevalence of anemia during pregnancy and others quite very low. In this regard, pregnant mothers from Somali, Dire Dawa, and Afar regions have high level and those from Addis Ababa and SNNPR as well as Tigray region had the lowest level of prevalence. Those regions with high prevalence of anemia have something in common; they are located in the eastern part of the country and are dominated by Muslim community. Another study in the area [10] has estimated a similar prevalence and identified that khat chewing and restrictive dietary behaviors are the key factors associated with the unacceptably high level of anemia among pregnant mothers [10]. On the other hand, the very low prevalence level of anemia in Addis Ababa should be interpreted very cautiously as only ten cases of pregnant women were included into the analysis. This is of course contrary to previous studies in the area [11, 24].

Contrarily to scientific plausibility [20] and other positive effects of electronic communications, pregnant mothers from households owning radio have shown a statistically inverse association with the occurrence of anemia during pregnancy. This might be attributable to the high level of adult illiteracy at rural communities whereby little comprehension of radio messages or lack of adequate and tailored information about pregnancy and pregnancy related issues in the national broadcasting services exists. Generally, as this is a new and unprecedented condition, further studies on the association between the two are demanded.

Unlike sociodemographic and household variables that exhibited little or no significant association with maternal anemia during pregnancy, most of the obstetric variables studied have either increased or reduced the risk of developing anemia during pregnancy. In this regard, pregnancy trimester, number of under five children in the household, previous history of abortion, breast feeding practices, and frequency of antenatal care visits showed a significant effect on anemia during pregnancy.

Pregnant mothers in the second or third trimester had a sevenfold raised risk of anemia during pregnancy compared to during the first trimester. This is consistent with several other local [8, 10] and international [15] similar studies. It is important, however, to interpret the results as every pregnancy has three trimesters but could only be classified once with the other variables.

Having too many under five children or too frequent birth is among the key predictors of anemia in Ethiopia identified by the current analyses. This is also consistent with the findings of other studies [14, 25–27] that limiting birth or using family planning to control and space births is a key contributing factor to the prevention of anemia during pregnancy. At the same time, women who experienced abortion or terminated pregnancy before the index pregnancy were also found to have a 2.63 times higher risk of developing anemia than those who did not (AOR, 2.63; 95% CI, 1.17–5.92). This is consistent with several other studies [28, 29].

The other important aspect of the findings is the fact that breast feeding patters of mothers contributed much to the occurrence of anemia during subsequent pregnancies. Women who have never breastfed and continued breastfeeding to the index pregnancy were almost eight and four times more likely to experience anemia during recent pregnancies compared to those who have ever breastfed (AOR, 7.64 and 4.05; 95% CI, 2.28–25.65 and 1.61, 10.91). This is not explained by any study so far which prompts further study on these associations and requires careful interpretation, as the same holds true for the selection of samples is not directly related to the current analysis.

The other obstetric characteristic with protective finding to the occurrence of anemia during pregnancy is the frequency of antenatal care visits. Pregnant mothers who had at least 3-4 and above four visits had a 51% and 64% less chance of developing anemia during pregnancy than those who had less or none at all (AOR, 0.49; 95% CI, 0.19–0.98, and AOR, 0.36; 95% CI, 0.08–0.64), respectively. On the other hand, despite lesser (20% compared to 24.8% and 37.1%) proportion, pregnant mothers who timely (<4 months) started antenatal care visits were found to be nonanemic compared to those who started late (4-5 months) and too late (6–9 months); the occurrence was not statistically significant in the final logistic regression model (AOR, 0.85 and 2.29; 95% CI, 0.42–1.69 and 0.82–6.38). This is also consistent with several other studies conducted locally [11, 14, 30] or globally [31–33].

*Limitations*. The study has a limitation of selection of study participants and some potential variables. This is to say that even if the data is collected from representative population across the country, it did not take anemia and its determinants into account to determine sample sizes and the data collection tool lacked considering variables affecting anemia. Therefore, caution should be taken while interpreting some results.

## 5. Conclusion and Recommendation

Generally, even if the prevalence of maternal anemia during pregnancy declined over time, still it remains a moderate public health problem in Ethiopia. The occurrence of anemia is mainly guided by several sociodemographic (maternal age and region), household (access to radio), and many of the obstetric characteristics including pregnancy trimester, number of under five children in the household, previous history of abortion, breastfeeding patterns (never or continued breastfeeding), and frequency of antenatal care visits.

Therefore, policy makers and other concerned program managers should focus on the key variables in their future planning to deal with the high level of anemia in the country. They should pay attention to older age, those in the eastern region of the country, second and third pregnancy trimesters, having too many under five children, women with previous history of abortion, those with infrequent (≤2) antenatal care visiting pregnant mothers in the programming, and implementation priorities. Further studies are also recommended to be conducted on why some special group of mothers (those who have access to radio and those from the eastern parts of the country) are at special risk of developing the problem without a clear implication to the risk.

## Figures and Tables

**Table 1:** Sociodemographic characteristics of pregnant mothers and their association with anemia during pregnancy in Ethiopia, EDHS 2011.

Sociodemographic characteristic	Anemic (#)/(%)	Nonanemic (#)/(%)	Crude OR (95% CI)	Adjusted OR (95% CI)
(1) Age				
15–19	5 (12.5)	35 (87.5)	1	1
20–24	45 (19.1)	190 (80.9)	1.64 (0.61, 4.38)	1.57 (0.57, 4.35)
25–29	110 (23.7)	355 (76.3)	2.13 (0.82, 5.53)	2.35 (0.87, 6.35)
30–34	67 (27.1)	180 (72.9)	2.56 (0.97, 6.75)	2.65 (0.97, 7.260)
35–39	29 (20.1)	110 (79.1)	1.79 (0.65, 4.96)	2.04 (0.71, 5.85)
40–44	12 (30)	28 (70)	3.07 (0.98, 9.64)	3.43 (1.04, 11.28)^*∗∗*^
45–49	2 (25)	6 (75)	1.82 (0.24, 13.70)	1.36 (0.15, 11.89)

(2) Residence				
Urban	25 (26.9)	68 (73.1)	1	1
Rural	245 (22.7)	836 (77.3)	0.77 (0.48, 1.25)	0.80 (0.43, 1.48)

(3) Region				
Tigray	16 (21.6)	58 (78.4)	1	1
Afar	7 (38.9)	11 (61.1)	2.23 (0.74, 6.71)	2.09 (0.68, 4.40)
Amhara	49 (31.2)	108 (68.8)	1.64 (0.86, 3.14)	1.62 (0.84, 3.14)
Oromia	129 (23.5)	419 (76.5)	1.11 (0.62, 1.99)	1.04 (0.57, 1.89)
Somali	29 (51.8)	27 (48.2)	3.93 (1.84, 8.39)	3.82 (1.71, 8.52)^*∗∗*^
Ben.-Gumuz	5 (27.8)	13 (72.2)	1.47 (0.47, 4.61)	1.32 (0.41, 4.25)
SNNPR	30 (10.6)	252 (89.4)	0.44 (0.22, 0.85)	0.41 (0.21, 0.82)^*∗∗*^
Gambella	1 (33.3)	2 (66.7)	1.79 (0.13, 23.8)	1.83 (0.11, 29.43)
Hareri	1 (33.3)	2 (66.7)	2.10 (0.14, 30.85)	2.10 (0.13, 32.89)
Addis Ababa	0 (0)	10 (100)		
Dire Dawa	3 (60)	2 (40)	4.97 (0.73, 33.64)	4.51 (0.63, 32.28)

(4) Maternal educ. status				
No education	206 (25)	617 (75)	1	1
Primary educ.	60 (18.4)	266 (81.6)	0.67 (0.48, 0.92)	0.95 (0.60, 1.51)
Sec. education	0 (0)	11 (100)	0.08 (0.002, 2.97)	0.24 (0.00, 10.82)
Higher educ.	5 (33.3)	10 (67.7)	1.31 (0.43, 4.01)	3.00 (0.66, 13.68)

(5) Maternal literacy level				
Cannot read/write	226 (23.7)	727 (76.3)	1	1
Partially read and write	25 (23.4)	82 (76.6)	0.99 (0.62, 1.59)	1.12 (0.61, 2.04)
Fully read and write	13 (13)	87 (87)	0.49 (0.27, 0.90)	0.46 (0.19, 1.11)

^*∗∗*^Significant at *P* value < 0.05, OR: odds ratio.

**Table 2:** Selected household characteristics of pregnant mothers and their association with anemia during pregnancy in Ethiopia, EDHS 2011.

Household characteristic	Anemic (#)/(%)	Nonanemic (#)/(%)	Crude OR (95% CI)	Adjusted OR (95% CI)
(1) Presence of electricity in the HH				
No	247 (23.7)	795 (76.3)	1	1
Yes	23 (19.7)	94 (80.3)	0.77 (0.47, 1.25)	0.73 (0.39, 1.38)

(2) Presence of radio in the HH				
No	154 (21)	578 (79)	1	1
Yes	116 (27.2)	311 (72.8)	1.39 (1.05, 1.84)	1.41 (1.01, 1.88)^*∗∗*^

(3) Presence of television in the HH				
No	260 (23.4)	849 (76.6)	1	1
Yes	10 (20)	40 (80)	0.83 (0.41, 1.68)	0.96 (0.39, 2.37)

(4) Access to safe water source				
Safe water sources	97 (22.1)	342 (77.9)	1	1
Unsafe water sources	174 (23.6)	562 (76.4)	1.09 (0.89, 1.45)	1.09 (0.81, 1.48)

(5) Sanitation facility				
Improved sanitation	**19 (25.3)**	**561 (74.7)**	1	1
Nonimproved sanitation	**251 (22.8)**	**348 (77.2)**	0.85 (0.49, 1.45)	0.81 (0.45, 1.43)

Total	271 (23)	904 (77)		

^*∗∗*^Significant at *P* value < 0.05, OR: odds ratio.

**Table 3:** Selected obstetric characteristics of pregnant mothers and their association with anemia during pregnancy in Ethiopia, EDHS 2011.

Obstetric (RH) characteristics	Anemic (#)/(%)	Nonanemic (#)/(%)	Crude OR (95% CI)	Adjusted OR (95% CI)
(1) Pregnancy trimester				
First	26 (11.3)	205 (88.7)	1	1
Second	140 (27.8)	364 (72.2)	3.07 (1.95, 4.84)	17.05 (3.69, 78.8)^*∗∗*^
Third	106 (24)	336 (76)	2.51 (1.58, 4.01)	17.71 (3.76, 83.27)^*∗∗*^

(2) Number of under five children				
1	146 (25)	439 (75)	1	1
2	86 (18.5)	379 (81.5)	0.68 (0.50, 0.92)	0.39 (0.18, 0.84)^*∗∗*^
3	39 (31.7)	84 (68.3)	1.40 (0.92, 2.14)	2.10 (0.52, 8.48)

(3) Ever had terminated pregnancy				
No	224 (22.6)	767 (77.4)	1	1
Yes	47 (25.5)	137 (74.5)	1.17 (0.81, 1.68)	2.63 (1.17, 5.92)^*∗∗*^

(4) Pregnancy intention				
Wanted (then or later)	231 (22.7)	786 (77.3)	1	1
Unwanted	39 (26.5)	108 (73.5)	1.24 (0.84, 1.84)	2.39 (0.98, 5.86)

(5) Practice of breast feeding				
Ever breastfeeding	233 (23.1)	744 (76.9)	1	1
Never breastfeeding	13 (26)	37 (74)	1.15 (0.6, 2.22)	7.64 (2.28, 25.65)^*∗∗*^
Continued breastfeeding	25 (21.9)	89 (78.1)	0.92 (0.57, 1.47)	4.05 (1.61, 10.91)^*∗∗*^

(6) Timing of first ANC visit				
Timely (<4 months)	28 (20)	112 (80)	1	1
Delayed (4-5 months)	31 (24.8)	94 (75.2)	1.29 (0.72, 2.36)	0.85 (0.42, 1.69)
Delayed much (4–9 months)	13 (37.1)	22 (62.9)	2.26 (1.01, 5.05)	2.29 (0.82, 6.38)

(7) Number of ANC visits				
No (0) visits	130 (23.3)	429 (76.7)	1	1
Low (1-2) visits	22 (31.4)	48 (68.6)	1.53 (0.89, 2.62)	1.09 (0.50, 2.36)
Adequate (3-4) visits	34 (26.4)	95 (73.6)	1.18 (0.76, 1.83)	0.49 (0.19, 0.98)^*∗∗*^
Frequent (4+) visits	16 (16.3)	82 (83.7)	0.64 (0.36, 1.13)	0.36 (0.08, 0.64)^*∗∗*^

Total	271 (23)	904 (77)		

^*∗∗*^Significant at *P* value < 0.05, OR: odds ratio.
